# Novel antibiotics: Are we still in the pre–post-antibiotic era?

**DOI:** 10.1007/s15010-015-0749-y

**Published:** 2015-02-21

**Authors:** R. Draenert, U. Seybold, E. Grützner, J. R. Bogner

**Affiliations:** Sektion Infektiologie, Medizinische Klinik und Poliklinik IV, Klinikum der Universität München, Pettenkoferstr. 8a, 80336 Munich, Germany

**Keywords:** Tedizolid, Ceftolozane, Omadacycline, Avibactam, Cadazolid

## Abstract

**Purpose:**

Therapeutic efficacy and safety in infections due to multidrug-resistant bacteria can be improved by the clinical development of new compounds and devising new derivatives of already useful antibiotics. Due to a striking global increase in multidrug-resistant Gram-positive but even more Gram-negative organisms, new antibiotics are urgently needed.

**Methods:**

This paper provides a review of novel antibiotic compounds which are already in clinical development, mainly in phase III clinical trials.

**Conclusion:**

Each of these new trials increases the possibility of new antibiotics receiving approval.

## Introduction

New antibiotics are urgently needed due to the alarming development of resistance against all antibiotics on the market and in clinical use [1, 2]. While this has been the case since the detection of antibiotics, recently the gap has widened due to the fact that new antibiotic drug classes have barely been introduced among others. Moreover, the prevalence of difficult to treat resistant and multi-resistant nosocomial organisms is rapidly increasing both for Gram-negative and Gram-positive bacteria. However, at the moment, Gram-negative bacilli are the greater threat. While the prevalence of methicillin-resistant *Staphylococcus aureus* (MRSA) has plateaued in many countries and is even declining in some, the frequency of enterobacteriaceae that exhibit high rates of antibiotic resistance including broad-spectrum beta-lactamases (extended-spectrum beta-lactamase, ESBL) and even carbapenemases is rapidly increasing and poses imminent threat for patients with infections due to these organisms. For this reason, a large number of long-known beta-lactam antibiotics based on penicillins or cephalosporins have become ineffective in many instances, even when combined with conventional inhibitors of beta-lactamases like clavulanic acid or tazobactam [1].

**Fig. 1:**
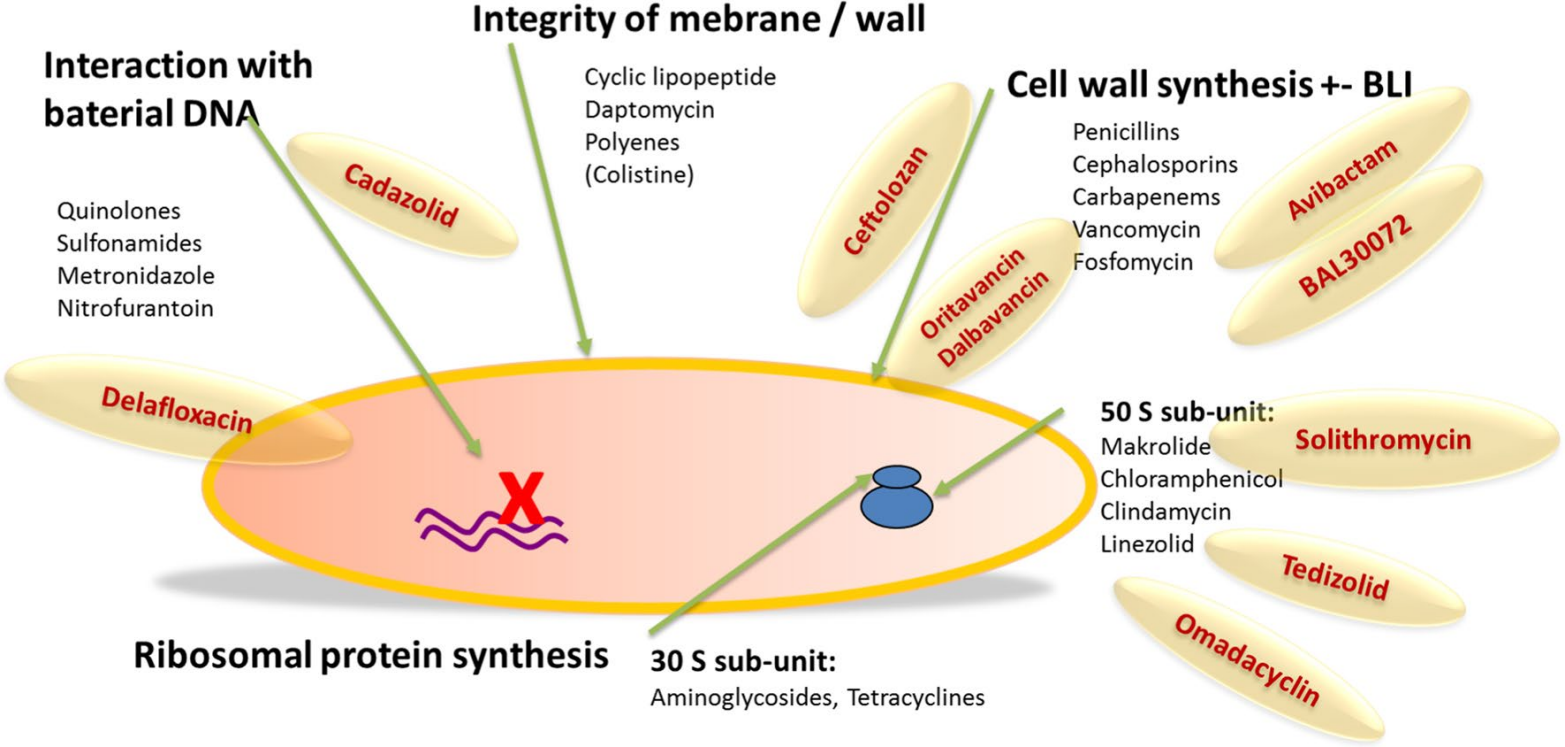
Schematic antibiotic action: new compounds in an overview

In the past couple of years, pessimism has been spreading with respect to the pipeline of new antimicrobials, and many pharmaceutical companies have declared that they will not continue to develop new antibacterial compounds. At present, the situation has changed positively, and those observing preclinical and clinical development strategies and activities have reason to be more optimistic. Several compounds have been developed in various drug classes and against resistant organisms in the whole spectrum of multidrug-resistant (MDR) bacteria (Fig. 1).

## Compounds against Gram-positive bacteria

### Novel long acting lipoglycopeptides oritavancin and dalbavancin

Acute bacterial skin and skin structure infections (ABSSSIs) are among the most frequent indications for antimicrobial therapy. The causative agents are usually Gram-positive bacteria including MRSA for which there are a number of treatment options that may be quite demanding with respect to application, dosing frequency, monitoring requirements, and duration of treatment. With the advent of novel lipoglycopeptides (in addition to teicoplanin, which has been available in Europe since 1992 and telavancin, which has received FDA approval in 2009), characterized by a concentration-dependent bactericidal activity and an extended elimination half-life, therapy of ABSSSI may become more easily manageable.

Oritavancin has (at least) three distinct mechanisms of action, namely inhibition of transglycosylation (like vancomycin), inhibition of transpeptidation (like beta-lactams), and disruption of cell membrane integrity (like telavancin) [3]. These result in rapid bactericidal activity against a number of Gram-positive pathogens. It also has a very long terminal half-life of >300 h [4] and demonstrated potent bactericidal activity of a single 1200 mg dose in an in vitro PK/PD model [5]. It is not metabolized, and there is no need for dose adjustment for renal or moderate hepatic impairment. This set of characteristics allows for very easy administration especially in an outpatient setting. Following a phase II study that did not support the daily administration of oritavancin [6], the recent phase III SOLO I trial involving 954 patients in the mITT population demonstrated non-inferiority of a single 1200 mg i.v. dose of oritavancin versus 7–10 days of twice daily i.v. vancomycin for ABSSSI with respect to all three efficacy end points including cure [7]. This held true for a variety of subgroup analyses. While nausea was somewhat more common in the oritavancin group (11 vs. 8.9 %), there was no statistically significant difference with respect to safety or tolerability in general. Oritavancin therefore has the potential to be used as single-shot treatment for ABSSSIs virtually eliminating adherence issues. As stated for the SOLO I as well as the SOLO II trial, the prolonged half-life of oritavancin was not associated with any safety issues including the 60 day follow-up period [7].

Dalbavancin is another semisynthetic lipoglycopeptide and has been evaluated for skin and soft tissue/skin structure infections [8, 9] as well as catheter-associated blood stream infections [10] already since the early 2000s. It has been shown to have only a minor impact on the gut flora [11]. Its terminal half-life of about 2 weeks [12] also allows for extended dosing intervals. Recently, the twin-phase III DISCOVER-1 and DISCOVER-2 trials for ABSSSI with 1312 patients in the pooled analysis were published. They demonstrated non-inferiority of two single doses of dalbavancin given 1 week apart compared to a standard twice-daily treatment regimen of i.v. vancomycin followed by an optional switch to p.o. linezolid for a total of 10–14 days [13]. This was true for both the primary end point of early clinical success and end of treatment success, independent of causative pathogen or comorbidity. Non-inferiority of a two-dose regimen is even more remarkable when considering that about half the patients met the criteria for systemic inflammatory response syndrome (SIRS). Dalbavancin had a favorable safety profile with both fewer adverse events and fewer patients experiencing adverse events. Nausea was noted to be the most frequent adverse event in the dalbavancin group occurring in 2.5 % of patients. Notably, the use of dalbavancin was associated with a significantly lower mortality (0.2 vs. 1.1 %). This has led to the FDA approval of dalbavancin for ABSSSI caused by *S. aureus* and *S. pyogenes* in May 2014.

### Tedizolid

Tedizolid phosphate is a new oxazolidinone compound. Its mechanism of action is similar to that of linezolid, which was the first drug in this class: It acts by inhibition of the ribosomal protein synthesis of bacteria. The exact mechanism of blockade is located at the 50S subunit of the bacterial ribosome, which is directly targeted by oxazolidinones in a way that inhibits binding of the tRNA by a conformational change in the binding moiety. Thereby, the protein synthesis chain is terminated [14, 15]. The active compound of tedizolid emerges by cleavage of the phosphate moiety of the prodrug. Tedizolid is active against Gram-positive bacteria, including MRSA. Vancomycin-resistant organisms and even linezolid-resistant MRSA have been found to be susceptible to the new drug [15–17].

Substantial pharmacological data on tedizolid show its suitability for clinical use as an antibiotic. Pooled analyses of intravenous and oral application have shown correlations of drug levels with clinical success. The absolute bioavailability of tedizolid is higher than 80 % which is comparable to linezolid [14, 17–20]. More positive pharmacological data show a lower interaction potential of tedizolid as compared to linezolid, e.g., no interaction with inhibitors of the monoamino-oxidase (MAO-inhibitors). The long half-life of tedizolid in combination with a high bioavailability offers the realistic rationale for once daily administration. With a 10- to 12-h half-life, drug levels of tedizolid above the MIC can still be accomplished at the end of a 24-h dose interval. About 80 % of the drug is eliminated via the gut and about 20 % renally.

Because the compound has good tissue penetration, one clinical area of use is ABSSSI.

The two-phase III ESTABLISH 1 and ESTABLISH 2 trials showed non-inferiority compared to linezolid, leading to approval of the new drug by the FDA in June 2014 [15, 16]. In ESTABLISH 2, a total of 666 patients with ABSSSI were recruited in nine countries with 58 participating treatment sites. A 1:1 randomization allocated patients in a double-blind manner to either tedizolid 200 mg intravenously followed by oral medication over 6 days or to linezolid at a dose of 600 mg by mouth twice daily over a period of 10 days. Stepdown from intravenous to oral medication could take place if the criteria of early clinical response were fulfilled. Early clinical response was defined as a reduction in the inflamed area by at least 20 % in the first 3 days. This criterion was reached roughly with equal frequency in both trial arms: In the tedizolid arm 85 % and in the linezolid arm 83 % of all patients had early clinical response. Regarding adverse events and safety issues, there was a significantly lower incidence of gastrointestinal adverse reactions in the tedizolid group (16 vs. 23 %) [16, 17, 21].

In addition to clinical efficacy data, there have been studies investigating pharmacological issues in special populations: Patients with renal failure and with hepatic insufficiency have been subject to pharmacokinetic sampling. The results show that no dose adjustment is necessary in patients with chronic renal failure with and without hemodialysis treatment. Only 10 % of the antibiotics were removed by dialysis. In patients with hepatic impairment, a moderate increase in drug levels in the range of 22–34 % was observed [19].

## Drugs against Gram-negative bacteria and broad-spectrum antibiotics

### BAL30072

BAL30072 is a monosulfactam antibiotic, which exhibits activity against carbapenem-resistant Enterobacteriaceae and non-fermenters [22–24]. The chemical structure of BAL30072 is related to that of aztreonam. BAL 30072 belongs to the group of beta-lactam antibiotic and inhibits bacterial cell wall synthesis. In vitro tests of combinations with beta-lactamase inhibitors such as clavulanate and BAL 29880 were described. The combination of BAL30072 with BAL29880 or clavulanate resulted in susceptibility rates of more than 90 % of all isolates tested in vitro. At 4 mg/l, BAL30072 showed activity against all OprD-deficient *Pseudomonas aeruginosa* [23–25]. Also, most *Acinetobacter baumannii* strains expressing OXA or NMD carbapenemases were susceptible to BAL30072 [25].

In an in vitro study, the activity of BAL30072 and other standard antibiotics against meropenem-resistant *A.* *baumannii* was evaluated, and a MIC90 of ≥64 was shown [23]. *Burkholderia pseudomallei*, the pathogen causing melioidosis, displays constitutive resistance toward a range of antimicrobials. Thus, treatment is difficult. In a study with its laboratory strains (1026b, 1710b) and several strains isolated in Thailand, more than 93 % of the isolates were susceptible to BAL30072 with minimal inhibitory concentrations (MICs) in the range of 0.004–0.016 µg/ml [24].

Compared to ceftazidim, meropenem, and imipenem, BAL30072 showed markedly higher activity with a MIC90 of 0.016 µg/ml. At the time of writing, BAL30072 is being investigated in phase I clinical studies [26, 27].

### Ceftolozane

Ceftolozane combined with tazobactam is being investigated for complicated urinary tract infections (cUTIs), complicated intra-abdominal infections (cIAIs) and ventilator-associated pneumonia (VAP). It is a novel cephalosporin with a structure similar to ceftazidim. It exerts activity against *Pseudomonas* (including ceftazidim-resistant *P. aeruginosa*) and also against bacteria-producing beta-lactamases such as TEM-1 (Temoneira: In 1965, this beta-lactamase was found in a patient called Temoneira) and SHV-1 (SHV = sulfhydryl variable) [28–30]. Ceftolozane shows no activity against bacteria-producing ESBL and carbapenemases. However, in combination with tazobactam, it is active against most of the bacteria-producing ESBL and against some anaerobes. The mean plasma half-life is 2.3 h, and protein binding of ceftolozane is low at approximately 20 %. Two-phase II studies of ceftolozane with tazobactam in urinary tract infections and abdominal infections have been completed. One-phase III study has been terminated since a larger phase III study in VAP was planned in comparison with meropenem which has already started to enroll. The FDA approved ceftolozane/tazobactam to treat adults cIAI and cUTI in December 2014, and approval in Europe is expected for the end of 2015 [31, 32]. So far, the clinical and microbiological activity appears comparable to imipenem and ceftazidim: For *P. aeruginosa,* the MIC was 0.5 mg/l for ceftolozane, 1 mg/l for ceftazidim, and 0.5 mg/l for imipenem [33–35].

### Delafloxacin (fluoroquinolone)

Quinolones are inhibitors of the bacterial DNA gyrase and topoisomerases (mostly topoisomerase IV). There are four generations of well-characterized quinolones which could be further developed, e.g., toward additional MRSA activity or better tissue penetration in neutral or acidic environments.

Delafloxacin is a promising investigational fluoroquinolone. In comparison with others, the substituent on position 7 of the quinoline ring system is not protonatable which results in a pKa shift. In contrast, other fluorquinolones are zwitterionic and therefore neutrally charged only at a physiological pH. Neutral charge is required for membrane penetration. Delafloxacin also permeates membranes at lower pH such as found in inflamed tissue [36, 37]. In inflammatory tissue of soft tissue infections, abdominal infections, or urinary tract infections, pH levels are mildly acidic (about 5.5–6). Under these conditions, e.g., 90 % of moxifloxacin is in a cationic state and therefore unable to permeate bacterial membranes. In contrast, delafloxacin is uncharged at this pH resulting in high cellular uptake [36]. Delafloxacin was already investigated in phase II studies for community-acquired pneumonia (CAP) and ABSSSI. The results were very promising so that phase III studies were initiated [38].

### Omadacycline and eravacycline

Tetracyclines are bactericidal and inhibit protein synthesis by binding to the 30S subunit of microbial ribosomes. While omadacycline and eravacycline do not have advantages over tigecyclin in terms of their microbiological activity, both drugs are orally bioavailable and therefore interesting for further development [15].

Currently, omadacycline is being compared to linezolid in a phase III study in ABSSSI. It is administered parenterally in the initial phase. After clinical recovery, a switch to oral administration is possible. Omadacycline doses are 100 mg for intravenous and 150 mg for oral application [38].

Phase III studies of eravacycline are conducted with the antibiotic comparators levofloxacin or ertapenem in urinary tract infections and cIAI. Eravacyclin is administered parenterally every 12 h in cIAI.

### Solithromycin (ketolide)

Solithromycin is a ketolide and derived from the macrolide erythromycin. The molecular mode of action is similar to telithromycin. It inhibits protein synthesis by binding ribosomal subunits. Microbiologically, the drug differs from macrolides due to additional MRSA activity and activity to resistant Streptococci.

Solithromycin is being investigated in clinical studies for CAP [39] and for gonococcal urethritis and compared with quinolones (levofloxacin and moxifloxacin) and the combination of ceftriaxone and azithromycin [38]. In vitro data and the existing clinical data suggest a lower risk of hepatotoxicity than for telithromycin [40].

### Novel beta-lactamase inhibitors

The beta-lactamase inhibitors currently available for clinical application, i.e., sulbactam, clavulanate and tazobactam, inhibit beta-lactamases of the molecular class A reflecting the original TEM and SHV genes. Recently, other beta-lactamases are emerging which contribute to the increasing resistance especially of Gram-negative bacilli. Among those are oxacillinases [belonging to class D; OXA-1/10 (OXA = oxacillin hydrolyzing capabilities)], cephalosporinases [belonging to class C; AmpC (ampicillin class C BLI)], CMY-2 (cephamycin-BLI no. 2), FOX-1 (cefoxitin-BLI) and the metallo-beta-lactamases [belonging to class B; IMP-1 (imipenem-BLI), VIM-1/2 (Verona integron-encoded metallo-beta-lactamase), NDM-1 (New Delhi metallo-beta-lactamase), CphA (metallo-beta-lactamase from *Aeromonas hydrophila*), and Sfh-1 (beta-lactamase from *Serratia fonticola*)]. Novel beta-lactamase inhibitors (e.g., diazabicyclooctane-related substances) are able to also inhibit enzymes of these other groups to a various degree. They therefore contribute substantially to meet the increasing need for new drugs against ESBL or Klebsiella pneumonia carbapenemases (KPCs)-producing bacilli. Important representatives of these new beta-lactamase inhibitors are avibactam and MK-7655.

### Avibactam

Avibactam belongs to the group of diazabicyclooctanes [22, 41]. It inhibits nearly all class A and class C (AmpC) beta-lactamases. Due to its activity against various beta-lactamases, it is suitable for clinical testing in the setting of severe infections with multi-resistant Gram-negative bacteria. It was combined with either ceftazidime or ceftaroline. In vitro, ceftazidime/avibactam is active against multi-resistant enterobacteriaceae [e.g., bacilli with inducible or derepressed AmpC-beta-lactamases, class A beta-lactamases, carbapenemases belonging to class A (KPC) and class D (OXA-48)]. However, metallo-beta-lactamase producers were resistant toward avibactam [42].

The clinical efficacy and the safety of ceftazidime/avibactam are being tested in phase III clinical trials with different clinical indications. Among these are urosepsis, severe urinary tract infections, and intra-abdominal infections. In phase II clinical trials, it was shown that the efficacy of ceftazidime/avibactam was non-inferior to the carbapenem control group [22, 43].

In the two-phase III RECLAIM-1 and RECLAIM-2 trials, intravenous ceftazidime/avibactam plus metronidazole was compared to meropenem for cIAI. Ceftazidime/avibactam (2000 mg/500 mg) was infused over 2 h. Meropenem (1000 mg) was given over 30 min. The combination ceftazidime/avibactam/metronidazole met its primary end point of non-inferiority as shown by preliminary results [43].

### Mk-7655

MK-7655 is a novel beta-lactamase inhibitor under clinical development. In vitro, it was tested against a KPC-2 carbapenemase-producing *Klebsiella pneumoniae* (isolate KP6339) and also against *P. aeruginosa* isolates lacking the OprD porin and overexpressing AmpC (isolates PA24226, PA24227, and PA24228). Synergism of MK-7655 in combination with imipenem was shown [44]. At the moment, a phase II study for severe urinary tract infections is being conducted. This study aims to proof that 125 or 250 mg MK-7655 in combination with imipenem is non-inferior to imipenem only [38].

### RPX7009 and AAI101

RPX7009 is a cyclic, boric acid-based beta-lactamase inhibitor active against serine-carbapenemases. It was given to 56 individuals in a dosage between 250 and 2000 mg as a single dose or up to 7 days as 3-h infusion. The substance is mainly eliminated via the renal pathway and did not accumulate. Analysis of tolerability showed no significant difference to placebo. Severe adverse events did not occur [45].

AAI101 is a hybrid ionic beta-lactamase inhibitor with broad activity against ESBL. Efficacy was shown in combination with ceftriaxone, cefepime, or piperacillin in animal models and was compared to combinations with tazobactam. Clinical efficacy was defined as lethality of an intraperitoneal application of ESBL Gram-negative bacteria, and efficacy was significantly higher for all three combinations with AAI101 than for the control groups [46].

## Cadazolid, a novel substance for treating *C. difficile*

Cadazolid is a novel antimicrobial agent with structural analogy to both oxazolidinones and fluoroquinolones. It has high and relatively selective efficacy against *C. difficile*. Its mechanism of action is inhibition of protein biosynthesis, which also leads to a highly effective suppression of toxin production. In addition, it strongly inhibits spore formation [47]. According to microbiological in vitro and animal models, one advantage of cadazolid is its relatively weak impact on the intestinal microbiota [48]. Also, there is no relevant intestinal absorption, leading to high intraluminal concentrations and a low systemic impact [49]. Cadazolid was tested in a multicentric, randomized, and double-blind phase II trial in patients with *C. difficile*-associated diarrhea (CDAD) using doses ranging from 250 to 1000 mg per day over 10 days [50]. Standard dose oral vancomycin was used as comparator. A total of 84 patients with CDAD were included, and the study was completed in December of 2012. The efficacy of all dosages of cadazolid was similar or better compared to vancomycin with respect to all clinical end points, specifically cure rate and frequency of relapses. At the same time, the drug was well tolerated. Currently, a dosage of 250 mg bid is being tested versus vancomycin 125 mg qid in the large phase III IMPACT trial [51].

## Conclusion

In conclusion, there are several promising compounds on their way through clinical development which will broaden the possibilities for treatment of MDR bacteria, especially against MRSA and Gram-negative ESBL bacteria. But the evolution of resistance mechanisms will not stop with the introduction of the new drugs. Therefore, the race must continue, and drugs with new mechanisms of action need to be investigated and tested for clinical use. Equally important, however, is the reduction in new infections and the counteraction of rising rates of antibiotic resistance with infection control mechanisms.
